# Case report of patient experience influenced by inadequate interactions between primary, secondary, and tertiary healthcare services in the south of Saudi Arabia

**DOI:** 10.1002/ccr3.2617

**Published:** 2019-12-22

**Authors:** Ibrahim M. Gosadi

**Affiliations:** ^1^ Department of Family and Community Medicine Faculty of Medicine Jazan University Jazan Saudi Arabia

**Keywords:** healthcare delivery, healthcare system, patient experience, patient satisfaction, social care

## Abstract

This report explains how health system failure can hinder provision of healthcare for a common health problem. Inadequate healthcare delivery can create barriers, lead to stress, prolong waiting time, and affect overall well‐being of patients.

## Graphical abstract

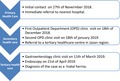

## 1 INTRODUCTION

This case report is describing the healthcare‐seeking process of a patient with heartburn in a region with the lowest patient satisfaction level in Saudi Arabia. In this report, we explain how health system failure can hinder provision of medical care for a common health problem and increase patient suffering.

The healthcare system in Saudi Arabia is composed of several public and private agencies, providing preventive and curative services on primary, secondary, and tertiary levels. A major provider of free healthcare services in Saudi Arabia is the Ministry of Health (MoH), which is contributing about 60% of hospital health services in the country. The Saudi MoH is currently facing challenges in provision of health services, resulting from the high demand induced by the free availability of health services, difficulties in access of healthcare, due to the large geography of the country, underutilization of electronic health services, and the lack of a nationwide electronic health system.^1^ These challenges are influencing patients’ experience and likely to affect the overall quality of healthcare.

The Saudi MoH has taken several initiatives to enhance the overall quality of provided health services. The 937 Call Center and Patient Experience Measurement Program are examples of Saudi MoH programs providing channels for healthcare seekers to interact with the MoH and give feedback concerning their experience. The Patient Experience Measurement Program is conducted by an independent third party to ensure valid measurement of patients’ experiences.^2^

The Patient Experience Measurement Program covers primary, secondary, and tertiary levels of health services provided by the Saudi MoH, such as primary health care centers (PHCCs), hospitals, and specialized centers, such as tumor and cardiac centers. The survey covers several components of the patient experience, including registration, transportation, medical staff, laboratory, radiology, and pharmacy services. On average, participating patients are asked to answer 30 questions depending on the type of service received.

The Saudi MoH is supervising 20 Directories of Health, operating in 13 administrative regions in the kingdom. In August 2018, the Saudi MoH revealed first findings of patients’ satisfaction survey covering the first half of the year 2018 with a total of 75 000 completed questionnaires. The average country‐wise patient satisfaction score in first half of 2018 was 67.3 where regional satisfaction scores varied between 73.9 and 54.5. In April 2019, the Saudi MoH revealed results of completed 59 000 questionnaires within the first quarter of the year 2019. The average country‐wise patient satisfaction score with Saudi MoH health services in the first quarter of 2019 was 72.2 where regional satisfaction scores varied between 76.8 and 63.4. In both reports, Jazan region scored the lowest patient satisfaction levels in comparison with other regions in the Kingdom.^2^

The findings of the patients’ satisfaction survey indicate that the low satisfaction of patients in Jazan is mainly driven by the quality of hospital services. Jazan is a peripheral region located more than 1000 Kilometers southwest of the capital and on the northern borders of Yemen. According to the latest census in Saudi Arabia, the number of citizens living in Jazan region is 1.1 million.^3^ The latest Saudi MoH yearly statistics book reported that the number of hospitals in Jazan region is 21. However, after the fire incident which resulted in several fatalities and injuries among admitted patients in Jazan General Hospital in the year 2015, the number of currently functioning hospitals is 20 where only one hospital acts as a tertiary referral hospital.^4^ Additionally, the demand for health services is further augmented by the war in Yemen, resulting in casualties treated in the region's hospitals.

It can be clearly observed that there are several challenges facing MoH concerning delivery of health services in Jazan region. The patient satisfaction survey is an evident indicator of reduction of quality of health service provision in Jazan region in comparison with other regions in the kingdom. However, there are several components influencing patients’ experience in Jazan that cannot be appropriately measured using quantitative methods. In this report, we attempt to describe the healthcare‐seeking process in Jazan region, starting from the primary healthcare level, going through the secondary healthcare facility and reaching a tertiary healthcare facility, using an active participation qualitative approach.

## 2 CASE PRESENTATION: HEALTHCARE‐SEEKING PROCESS

This report is based on the active participation of the researcher [IG] as a patient seeking healthcare from Saudi MoH healthcare facilities in Jazan region. In 2018, IG suffered heartburn for several months with one episode of dysphagia that lasted for few days and resolved spontaneously. Heartburn can be a symptom of Gastroesophageal Reflux Disease (GERD). The prevalence of GERD is reported to reach 28.7% among the adult Saudi population.^5^ This was a valuable opportunity to investigate factors explaining why Jazan region health services scored the lowest satisfaction levels among patients in the region.

**Figure 1:**
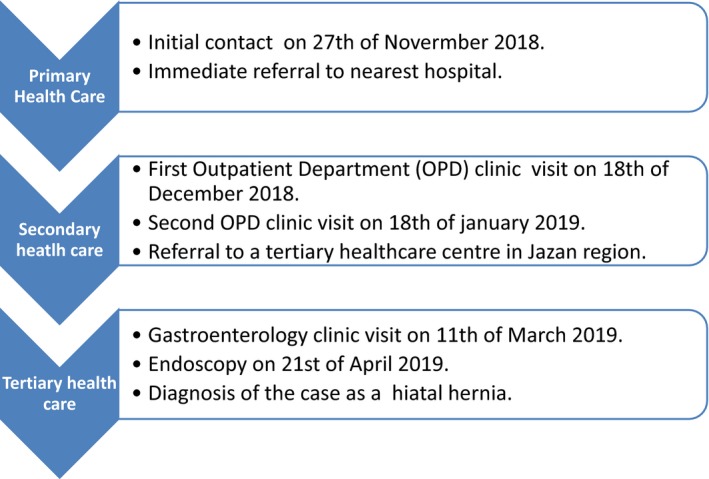
Timeline of healthcare‐seeking process in primary, secondary, and tertiary healthcare facilities in Jazan region, Saudi Arabia

In this report, a qualitative approach was utilized to identify shortcomings in provision of health services, where the patience of the investigator is a cornerstone of acquiring this experience. This report summarizes a healthcare‐seeking process that lasted for 5 months between November 2018 and April 2019 in multi‐level MoH health facilities in Jazan region (Figure 1). IG is a consultant epidemiologist with experience in conducting qualitative investigations involving collection and analysis of qualitative data. The process of this investigation involved active participation of the researcher in this patient experience, informal interviews and interactions with physicians and other healthcare employees in their natural work settings, recording detailed field notes and utilization of online and calls services provided by the MoH. IG is a native Arabic speaker and fluent in English, which enabled interaction with both Arabic and non‐Arabic healthcare staff at visited MoH facilities.

### 2.1 Primary healthcare

The initial contact of IG with a Saudi MoH facility was made on 27th November, 2018 via visiting the nearest primary healthcare center (PHC). Although IG has a medical record at the visited PHC, the medical record number was not retrieved. IG, as a healthcare seeker, was not aware that the Saudi MoH has established a new Central Appointment System called Mawid, which was designed to enable reserving appointments before visiting PHCs which, somehow, reduced the need to retrieve the previously produced medical record number.^6^ The Mawid system utilizes the National Identification Number of Saudis as a mandatory requirement for the registration and uses texting of message via mobile phone as a one‐way channel of communication with patients.

Due to lack of awareness about the new appointment system, the visit to the PHC took more than one hour where most of the time was dedicated to resolving the registration of IG in the Mawid system. PHC staff explained that no patient is allowed to receive health services at the center without booking an appointment via this system. However, it was clearly apparent that the provided electronic devices and internet connection at the visited PHC were repeatedly failing to complete the registration, which actually increased the time spent in the center.

After completion of registration and the appointment booking process, IG was able to be seen by a male Arabic‐speaking general physician on the same visit to PHC. The time spent with the physician was limited where minimal medical history was taken, and no physical examination was made. Additionally, no laboratory investigations were requested. The PHC physician preliminarily diagnosed the case as GERD and prescribed 20 mg of Omeprazole to be taken on a daily basis. IG was referred to the nearest hospital to seek further medical advice from specialists in internal medicine.

Using the Mawid system, PHC staff were able to inquire about the nearest available appointment at the internal medicine clinic at a nearby hospital. The nearest available appointment at the secondary hospital was found to be 21 days after the initial visit to the PHC. IG was able to receive a confirmatory mobile text message from the Mawid system, indicating details of the hospital appointment, such as date, time, and clinic name.

### 2.2 Secondary healthcare

On 18th December 2018, IG visited the referral secondary hospital, where the appointment at the internal medicine clinic was at 10:30 AM. After proceeding to the Outpatient Department (OPD) reception, IG was advised to open a new medical record at the hospital and was asked to visit the medical record unit at the hospital. The process of opening a new medical record at the hospital was not clear and took about one hour to be completed. After creating a new medical record, IG was able to be seen by the internal medicine physician. However, the waiting time was three hours, which indicates a lack of organization in the appointment system as it differs from the scheduled time.

It was noted that the hospital had an electronic system, where the physician was able to access the patients’ history and findings of medical examination, request laboratory and radiological investigations and prescribe medications. However, the Arabic‐speaking internist was apparently new at the hospital and was not able to request specific tests, such as Helicobacter pylori and advised IG to have the test performed at private laboratories. Inability of the internist to find and request needed laboratory investigations via the hospital's electronic system may suggest lack of orientation of hospital's physicians about the utilized electronic system. This lack of orientation is likely to hinder adequacy of physicians practice, needlessly prolong time spent with each patient and eventually increases the waiting time of patients at clinic.

History taking and physical examination were limited, and only blood pressure was taken. The total time spent with the internist was approximately 10 minutes where the medical advice was to cease taking Omeprazole for 1 month and requested routine complete blood count and blood chemistry. Afterwards, the internist was not able to provide a preliminary diagnosis and requested a follow‐up session after 1 month for further evaluation of the case.

The follow‐up session was scheduled at 1 pm on 18 January 2019. Upon the second follow‐up visit to the hospital, time of arrival at the hospital was recorded by the receptionist. The waiting time to see the physician was less than an hour. In the follow‐up session, IG was seen by a different Arabic‐speaking female internist. After a brief history taking and no physical examination, the internist explained, in clear Arabic language, the nature of the condition and described several lifestyle modifications to alleviate the condition, such as avoiding consumption of carbonated soft drinks, increasing consumption of fruits and vegetables, and to avoid lying down for a minimum of three hours after consumption of meals.

The internist who was seen at the follow‐up session did not request any further laboratory investigations and prescribed a daily dose of 40 mg of Omeprazole where the prescription was administered electronically via the hospital system enabling a paperless collection of medication from the pharmacy. Additionally, the internist referred IG to the only tertiary hospital in the region for further evaluation and possibility of performing upper gastroesophageal endoscopy.

In this occasion, referral to the tertiary hospital was not possible via the Mawid system, where the internist had to hand‐write a referral letter. IG was advised to visit the OPD manager for further assistance in completion of the referral. The OPD administration office was crowded with visitors and lacked organization. IG was asked to provide his national ID card and the referral letter where, apparently, the ID card and the referral letter were scanned and sent via email to the tertiary hospital. OPD administration staff asked IG to leave and wait for a mobile text concerning his appointment at the tertiary hospital.

Although IG was informed that he would receive a mobile text message detailing his appointment at the tertiary hospital, no message was received concerning this appointment. Additionally, it was not possible to enquire about this appointment via the Mawid system. IG contacted the MoH call service (937) where the responding staff at the ministry advised IG to visit the hospital to inquire about the appointment. It appeared that online and call services were not sufficient to solve this issue of failed communication.

After waiting for 20 days, IG had to visit the secondary hospital OPD to inquire in person about the referral. It appeared that an appointment was made; however, OPD staff at the secondary hospital failed to communicate the appointment information to IG. This indicates a lack of uniformity of the utilized communication system among different hospitals in the MoH.

### 2.3 Tertiary healthcare

The nearest available appointment at the tertiary hospital was 52 days after the referral decision making. This indicates both how congested the health service was at the only tertiary hospital in the region and a difficulty of access. Upon visiting the tertiary hospital on 11 March 2019, it appeared that there was no need to attend in person to open a new medical record as it was created by the medical record department, utilizing the information shared from the secondary hospital. This notion provides an evidence of procedural inconsistencies in registration of patients between the region hospitals.

Waiting time at the tertiary hospital gastroenterology clinic was less than an hour on the scheduled appointment time. On this occasion, the attending doctor was an Arabic‐speaking Saudi male consultant gastroenterologist. Although the referral letter was sent via email, it was noted that the electronic health system utilized at the tertiary hospital was not able to provide the referral information to the gastroenterologist. After a minimal history taking and no physical examination, the treating gastroenterologist questioned the importance of this referral and critiqued the current workload at the tertiary hospital due to unnecessary referrals from primary and secondary health facilities at the ministry.

The management plan was changed via the consultant gastroenterologist, when Omeprazole was stopped and a daily dose of 20 mg of Domperidone and a dose of 40 mg of Esomeprazole were prescribed electronically, facilitating a paperless collection of medication from the pharmacy. To investigate the cause of dysphagia in IG's condition, an appointment to perform an upper gastroesophageal endoscopy was made after 41 days. It was noted that the endoscopy appointment was made at the clinic, and the receptionist was visited to print the appointment letter. The attending nurse at the clinic provided a letter of instructions needed to facilitate performing the endoscopy.

On 21st April, IG attended the endoscopy department half an hour before the scheduled endoscopy at 8 am. It was noted that the female nurse who was responsible for admission of patients to the endoscopy clinic was not able to speak Arabic, which hindered the communication process with the attending Saudi patients. IG had to communicate in English with the attending nurse, when she asked whether IG had had the laboratory investigations needed before endoscopy. This was confusing as no laboratory investigations had been requested by the treating gastroenterologist.

Due to the possibility of sedation to perform the endoscopy and subsequent risk posed by self‐driving, the nurse inquired about availability of an accompanying individual. Driving or taking a taxi are the only means of attending the hospital, where no public transportation services are available in Jazan region indicating presence of difficulty of access to healthcare. IG is living in a remote rural area where finding a taxi is difficult. This made accessibility to perform the endoscopy more difficult, as an accompanying relative who was able to drive had to attend the endoscopy session with IG.

After waiting for nearly 3 hours, IG was admitted to the endoscopy room. Some of the attending nurses at the endoscopy room were Arabic speakers, and they were able to provide clear instructions for the endoscopy during preparation. The endoscopy was performed by the same consultant gastroenterologist who admitted the case. According to the findings of the endoscopy, IG's condition was diagnosed as a mild hiatal hernia requiring no further surgical procedures, and continuation on the pharmacological plan was advised.

## 3 DISCUSSION

This report is a detailed patient experience of a health‐seeking process in a region with low, country‐wise, patient satisfaction levels. We were able to identify several health service provision issues that may explain the low patient satisfaction scores in Jazan region. These issues are related to the possible limited role of PHCs, leading to prolonged waiting times and congestion of secondary and tertiary healthcare levels, improper utilization of health informatics systems, lack of communication between different levels of healthcare in the region, and variation in clinical practice of physicians.

There are several investigations conducted in Saudi Arabia to assess quality of healthcare in different settings.^7^, ^8^, ^9^, ^10^ However, none were conducted in Jazan region. A systematic review assessing quality of healthcare in PHCs in Saudi Arabia reported a substantial variation in quality of healthcare among PHCs in Saudi Arabia.^11^ Furthermore, management and organization of primary care services was indicated as an important factor in improving quality of healthcare.^11^

In a recent systematic review assessing patients’ satisfaction with PHCs services in Saudi Arabia, it was revealed that the mean overall patient satisfaction rating was 84% [Standard deviation: 7.3%]. However, the review concluded the presence of a contradiction between patients measured experience and their overall satisfaction where certain factors, such as income and education levels, may result in overestimation of satisfaction.^12^ Additionally, in a study assessing nonurgent presentations to hospitals' emergency departments in Saudi Arabia, it was noted that 65% of a sample of 350 patients were nonurgent cases who favored to attend emergency departments rather than PHCs.^13^

The lack of an organized health delivery system is likely to influence the adequacy of physicians’ practice and the adequacy of clinical care. For example, none of the physicians involved in this patient experience asked IG about his occupation, suggesting inadequacy in history taking. In a Norwegian study assessing failure of treatment of GERD, it was reported that failure of interaction of physicians between different levels of healthcare was a leading contributor in treatment failure.^14^ This was similar to what was observed in this patient experience where there was no interaction between physicians even between physicians in the same hospital. Additionally, inconsistencies in communicating medical records between different healthcare levels were noted where PHC referral note was hand‐delivered to physicians in secondary healthcare hospital upon arrival while referral note from secondary healthcare hospital was scanned and sent via email to the tertiary healthcare hospital.

This report was helpful in generating further questions concerning delivery of healthcare service in Jazan region that are worth investigating. Are patients and healthcare staff satisfied with the online appointment system and MoH call service? Why isn't the online appointment system connected to tertiary healthcare levels? Are physicians in secondary and tertiary healthcare levels in Jazan region satisfied with the referral system? What are the factors which hinder communicating clinical data between physicians in different levels of health care in Jazan region? Is there a difference in the medical records system between hospitals in Jazan region? Is excessive referral from PHCs responsible for delay of receiving healthcare from secondary and tertiary healthcare facilities in Jazan region? Is the practice of physicians in different levels of healthcare in Jazan region an evidence‐based practice? Are physicians in secondary and tertiary healthcare facilities satisfied with the electronic medical systems operating in hospitals? How often do physicians in secondary and tertiary healthcare settings engage in provision of lifestyle modification advice for their patients?

Answering these questions requires engaging patients, healthcare staff, and health officials in the Directory of Health in Jazan region in multiple subsequent qualitative and quantitative investigations. Additionally, there are several practical solutions that may enhance the overall patients experience in Jazan region such as equipping primary and secondary healthcare levels with necessary staff and infrastructure to manage common cases and reduce unnecessary transfers to tertiary healthcare level, upgrade of Mawid system to involve facilitating transfers to tertiary healthcare services, finally, developing an electronic system to enable sharing clinical notes between physicians in different healthcare levels and to ensure uniformity of utilized medical records systems in different health facilities.

## CONFLICT OF INTEREST

The author declare that there is no competing interests.

## AUTHOR CONTRIBUTIONS

IG was responsible for all steps pertaining to preparation of this manuscript.

## ETHICS APPROVAL AND CONSENT TO PARTICIPATE

Not applicable (all procedures described were carried out as a part of standard care and no patients other than the author were involved).

## References

[1] Almalki M , Fitzgerald G , Clark M . Health care system in Saudi Arabia: an overview. East Mediterr Health J. 2011;17(10):784‐793. 22256414 10.26719/2011.17.10.784

[2] Saudi Minstry of Health . Patient experience measurement program. 2019 https://www.moh.gov.sa/en/Ministry/pxmp/Pages/default.aspx.Accessed June 20, 2019.

[3] General Authority for Statistics .The general population and housing census. 2010 https://www.stats.gov.sa/en/13.Accessed June 27, 2019.

[4] Saudi Ministry of Health .Annual statistical book. https://www.moh.gov.sa/en/Ministry/Statistics/book/Documents/ANNUAL-STATISTICAL-BOOK-1438H.pdf. Published 2017.Accessed February 2, 2019.

[5] Alsuwat OB , Alzahrani AA , Alzhrani MA , Alkhathami AM , Mahfouz MEM . Prevalence of gastroesophageal reflux disease in Saudi Arabia. J Clin Med Res. 2018;10(3):221‐225. 29416581 10.14740/jocmr3292w PMC5798269

[6] Saudi Minstry of Health . (Mawid) Service. 2019 https://www.moh.gov.sa/en/eServices/Pages/cassystem.aspx.Accessed June 27, 2019.

[7] El‐Gilany AH , Aref Y . Failure to register for antenatal care at local primary health care centers. Ann Saudi Med. 2000;20(3–4):229‐232. 17322663 10.5144/0256-4947.2000.229

[8] Al‐Khaldi YM , Al‐Sharif AI . Availability of resources of diabetic care in primary health care settings in Aseer region. Saudi Arabia. Saudi Med J. 2002;23(12):1509‐1513. 12518203

[9] Al‐Khaldi YM , Al‐Sharif AI , Al‐Jamal MN , Kisha AH . Difficulties faced when conducting primary health care programs in rural areas. Saudi Med J. 2002;23(4):384‐387. 11953760

[10] Aljasir B , Alghamdi MS . Patient satisfaction with mobile clinic services in a remote rural area of Saudi Arabia. East Mediterr Health J. 2010;16(10):1085‐1090. 21222426

[11] Al‐Ahmadi H , Roland M . Quality of primary health care in Saudi Arabia: a comprehensive review. Int J Qual Heal care J Int Soc Qual Heal Care. 2005;17(4):331‐346. 10.1093/intqhc/mzi046 15883128

[12] Senitan M , Alhaiti AH , Gillespie J . Patient satisfaction and experience of primary care in Saudi Arabia: a systematic review. Int J Qual Heal care J Int Soc Qual Heal Care. 2018;30(10):751‐759. 10.1093/intqhc/mzy104 29860320

[13] Alyasin A , Douglas C . Reasons for non‐urgent presentations to the emergency department in Saudi Arabia. Int Emerg Nurs. 2014;22(4):220‐225. 24703789 10.1016/j.ienj.2014.03.001

[14] Farup PG , Blix I , Forre S , et al. What causes treatment failure ‐ the patient, primary care, secondary care or inadequate interaction in the health services? BMC Health Serv Res. 2011;11:111. 21599926 10.1186/1472-6963-11-111 PMC3126699

